# Healthcare workers’ views on decentralized primary health care management in Lesotho: a qualitative study

**DOI:** 10.1186/s12913-024-11279-3

**Published:** 2024-07-11

**Authors:** Ermyas Birru, Melino Ndayizigiye, George Wanje, Tholoana Marole, Patrick D. Smith, Masebeo Koto, Ryan McBain, Lisa R. Hirschhorn, Mathabang Mokoena, Annie Michaelis, Joel Curtain, Emily Dally, Afom T. Andom, Joia Mukherjee

**Affiliations:** 1 Partners in Health, House No. 233, Cnr. Lancers & Caldwell Rd, Maseru West, Private Bag A391, Maseru, 100 Lesotho; 2 Department of Global Health, University of Washington School of Public Health, Seattle, WA USA; 3 Department of Community Health and Prevention, Drexel University Dornsife School of Public Health, Philadelphia, PA USA; 4 Ministry of Health and Social Welfare, Maseru, Lesotho; 5 Division of Global Health Equity, Brigham and Women’s Hospital, Boston, MA USA; 6 Havey Institute for Global Health – Ryan Family Center for Global Primary Care, Feinberg School of Medicine, Northwestern University, Chicago, IL USA; 7 Partners in Health, Boston, MA USA; 8 Department of Global Health and Social Medicine, Harvard Medical School, Boston, MA USA

**Keywords:** Decentralization, Health systems research, Primary health care, Primary health care management, Health care reform, Decision making, Evidence-based policy, Implementation, Qualitative research, Impact, Organizational change

## Abstract

**Background:**

Lesotho experienced high rates of maternal (566/100,000 live births) and under-five mortality (72.9/1000 live births). A 2013 national assessment found centralized healthcare management in Ministry of Health led to fragmented, ineffective district health team management. Launched in 2014 through collaboration between the Ministry of Health and Partners In Health, Lesotho’s Primary Health Care Reform (LPHCR) aimed to improve service quality and quantity by decentralizing healthcare management to the district level. We conducted a qualitative study to explore health workers’ perceptions regarding the effectiveness of LPHCR in enhancing the primary health care system.

**Methods:**

We conducted 21 semi-structured key informant interviews (KII) with healthcare workers and Ministry of Health officials purposively sampled from various levels of Lesotho’s health system, including the central Ministry of Health, district health management teams, health centers, and community health worker programs in four pilot districts of the LPHCR initiative. The World Health Organization’s health systems building blocks framework was used to guide data collection and analysis. Interviews assessed health care workers’ perspectives on the impact of the LPHCR initiative on the six-health system building blocks: service delivery, health information systems, access to essential medicines, health workforce, financing, and leadership/governance. Data were analyzed using directed content analysis.

**Results:**

Participants described benefits of decentralization, including improved efficiency in service delivery, enhanced accountability and responsiveness, increased community participation, improved data availability, and better resource allocation. Participants highlighted how the reform resulted in more efficient procurement and distribution processes and increased recognition and status in part due to the empowerment of district health management teams. However, participants also identified limited decentralization of financial decision-making and encountered barriers to successful implementation, such as staff shortages, inadequate management of the village health worker program, and a lack of clear communication regarding autonomy in utilizing and mobilizing donor funds.

**Conclusion:**

Our study findings indicate that the implementation of decentralized primary health care management in Lesotho was associated a positive impact on health system building blocks related to primary health care. However, it is crucial to address the implementation challenges identified by healthcare workers to optimize the benefits of decentralized healthcare management.

**Supplementary Information:**

The online version contains supplementary material available at 10.1186/s12913-024-11279-3.

## Background

Decentralization of healthcare management entails the transfer of decision-making authority and resources from central government authorities to regional or local levels in low- and middle-income countries (LMICs) [1–5]. Decentralized healthcare management (administration) aims to improve the efficiency, effectiveness, and responsiveness of health services by bringing decision-making closer to the communities and patients they serve [6, 7]. Decentralization can involve changes to management of political, administrative, and fiscal systems, and frequently encompasses the transfer of responsibilities such as management of health care personnel, staff recruitment, procurement of supplies and equipment, as well as procurement and delivery of services [8, 9]. It is recognized as a potential solution for addressing challenges encountered by central governments in delivering quality health services to underserved or remote areas in LMICs. Past research has shown promises numerous benefits of decentralization, including improved resource allocation, heightened community participation and ownership of healthcare service delivery and enhanced responsiveness of health services to local needs [1, 10].

However, some studies have demonstrated that decentralization has not resulted in expected improvements in healthcare delivery and that there remains variation in service quality, availability of essential drugs and worker motivation across decentralized sites [11–13]. These studies have associated the lack of impact of decentralization with various factors and implementation strategies including insufficient funding, inadequate stakeholder engagement, ambiguous autonomy for local and regional level management, and a lack of appropriate infrastructure. Additionally, poor planning and coordination among decentralized entities may lead to worsening of fragmented healthcare systems and inefficiencies [14]. Other studies suggest that existing inequities may be reproduced in decentralized sites by decision makers, depending on existing priorities of equity in management decision, and a lack of clarity of the role of the district management team [15, 16]. There is a paucity of studies describing health workers’ experiences in a decentralized system in Lesotho. Understanding such perspectives is important as healthcare workers are the primary actors providing and managing care and services at the frontline [16].

## The Lesotho Primary Health Care Reform (LPHCR)

Lesotho experienced multiple health challenges, including high rates of maternal (566/100,000 live births) and under-five mortality (72.9/1000 live births), a high prevalence of HIV/AIDS (22.7 percent among individuals aged 15 years or older), and an estimated tuberculosis prevalence of 581 per 100,000 population for those ≥ 15 years in 2019, all compounded by restricted access to essential health services in rural areas [17–23]. Despite being an original member country that ratified the Alma Ata declaration in 1978 [24], the impact of the HIV pandemic on Lesotho, the country with the second-highest HIV prevalence globally in 2013 stymied progress toward achieving the promises of Alma Ata. An assessment conducted in 2013 found that healthcare management and decision-making were primarily centralized within the central Ministry of Health, resulting in fragmented and ineffective management in district health teams (refer to Supplement 1.1) [25, 26]. As a result, an external consulting group commissioned by the Government of Lesotho made a recommendation to reform the provision of primary healthcare to better serve the needs of the people in Lesotho. Launched in 2014, in collaboration between the Ministry of Health and Partners In Health (PIH), the Lesotho Primary Health Care Reform (LPHCR) aimed to enhance the quality and quantity of service delivery and improve primary health care in Lesotho. The LPHCR had three main components: decentralizing healthcare management to the district-level team, improving the coverage and quality of service delivery through better medicines and staffing to address the disease burden, and enhancing the linkage between community programs and the broader healthcare system by strengthening management authority at the subnational levels. The LPHCR initiative was launched with support from PIH, a global non-profit organization that collaborates with governments to provide care and strengthen public health systems [27] and other partners. The initiative aimed to rebuild a comprehensive primary healthcare system, initially piloting in four out of the total ten districts, including 70 primary health care facilities [28, 29].

Prior to Lesotho primary health care reform implementation, the health services delivery had gaps at different levels. At community level, many villages had no village health workers and there was no mechanism of knowing what those that existed were doing as there were no reporting structure for them [28]. The health centers lacked enough human resources and were not regularly receiving supportive supervisions to ensure adherence to national guidelines and protocols. Furthermore, health centers were equipped to provide certain essential health services such as conducting deliveries which was one of the main causes of home deliveries that resulted in a high maternal and neonatal mortalities. The district hospitals had different reporting structure than health centers. The hospitals were reporting to the Director General of Health Services while health centers were reporting to the district public health nurse [28, 30]. As part of the LPHCR rollout, decision-making power, supply chain management, and human resource management were transitioned from the central Ministry of Health to the district health management team. At the health facility level, health center committees were established, consisting of community representatives, along with additional staff such as Village Health Worker Coordinators, cooks, and extra nurses. Another change involved the implementation of monthly joint supportive supervision and mentorship at each health facility by district health management team members and PIH’s technical team. The focus of this supportive supervision was comprehensive primary healthcare rather than on one specific disease or program. Stakeholders envisioned an improved health system in Lesotho using the World Health Organization’s (WHO) six health systems building blocks framework (service delivery, health information systems, access to essential medicines, health workforce, financing, and leadership/governance), aiming to enhance community participation and ownership in healthcare service delivery while strengthening the health system’s capacity for planning, monitoring, and evaluation [31]. With LPCHR, all public health and clinical service provisions in the pilot districts are now under the district health management team (refer to Supplement 1.1 and 1.2).

This study documented healthcare workers’ perspectives on decentralization, outlining successes and challenges to inform future implementations for enhancing the quality and quantity of primary healthcare delivery. The paper also described the restructuring of the district health management team (DHMT), a key aspect of decentralizing primary health care management in Lesotho. The objectives of this study were to provide valuable insights for understanding and refining existing decentralized models in Lesotho and similar settings. We conducted key informant interviews (KIIs) to explore healthcare workers’ perspectives on the role of decentralized primary health care management in enhancing the six-health system building blocks in Lesotho.

## Methods

### Study setting

**Fig. 1:**
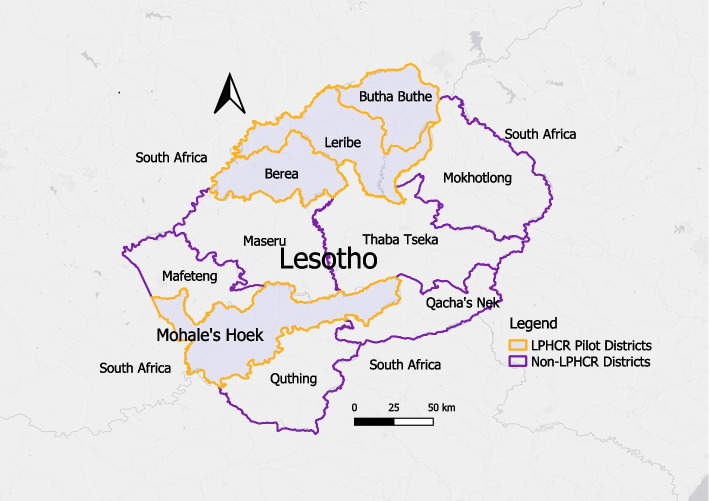
Location of Lesotho Primary Health Care Reform (LPHCR) pilot districts. QGIS (Quantum Geographic Information System) version 3.16 Hanover, a free and open-source software publicly available, was employed to create the maps. The background basemap was sourced from OpenStreetMap via the QGIS software. District and national boundary shapefiles were obtained from the Humanitarian Data Exchange website, which is publicly accessible at https://data.humdata.org/dataset/cod-ab-lso. This map was created by one of the authors (EB)

Lesotho, a mountainous country within South Africa, comprises an estimated population of 2 million, spread across 10 districts in four agro-ecological zones: lowlands, foothills, the Senqu River valley, and highlands [32, 33]. Health expenditure accounted for 12.7% of the 2018–2019 national budget, slightly below the 15% target set by the Abuja declaration [34, 35]. The Lesotho Primary Health Care Reform (LPHCR) was implemented from July 2014 to June 2017 in four government-selected districts, collectively representing 40% of the population—Berea, Butha Buthe, Leribe, and Mohale’s Hoek (Fig. 1) [28, 29, 32]. The Lesotho’s health system includes private health facilities, Christian Health Association of Lesotho (CHAL) facilities and public health facilities. This study included public health and CHAL facilities, which are supervised by District Health Management Teams (DHMT) that oversee and coordinate health service delivery in the districts.

## Study design

We conducted a qualitative study employing semi-structured KIIs (refer to Supplement 2), to interview health care workers (HCW) representing the different levels of Lesotho’s health system, including the central Ministry of Health, district health management teams, health facilities, and communities in the four pilot districts of the LPHCR initiative. The interview guides were developed using the WHO health system building blocks framework, which include service delivery, health information systems, access to essential medicines, health workforce, financing, and leadership/governance [31]. During the intervention design, the team considered the health system building blocks as the most suitable framework, given the project’s focus on health system strengthening [36, 37]. Interview questions were designed to elucidate HCWs understanding of the LPHCR’s ultimate goals and targets, as well as perceived benefits and challenges and successes, in implementation. Participants were purposively selected from the four pilot districts of the LPHCR initiative, as well as the central ministry of health office. Participants were prompted to share their experiences and opinions regarding the LPHCR.

### Data collection and sampling

From August 2017 to January 2018, we conducted a qualitative study employing two trained qualitative data officers—one female Mosotho fluent in both Sesotho and English, and one non-Mosotho English speaker. We used a stratified design to interview members for specific roles (*n* = 5) in each of the 4 districts, with a total of 20 interviews and five director level officials from the central ministry of health. Participants were recruited through emails and telephone calls, and a pre-established script was used to gauge their interest. Upon confirmation, in-person interviews were scheduled, and participants provided informed written consent. The interviews, lasting 60–90 min, were conducted in English or Sesotho based on the participant’s preference, in confidential settings, and were audio-recorded. Qualitative data officers took field notes during the sessions. To ensure no bias favoring the intervention, the qualitative team operated semi-independently with only funding and administrative support provided by the implementing agencies.

### Data analysis

Audio-recorded interviews were transcribed verbatim in Sesotho or English by the two data officers. Sesotho text was translated verbatim into English. Translated transcripts were reviewed with the audio files for literal meaning. Data were analyzed primarily using a directed content analysis approach guided by the WHO building blocks framework domains since our focus was on health systems strengthening, with a combination of limited conventional content analysis to enable us to contextualize factors within this target group [38]. We employed both deductive and inductive approaches in codebook development and analysis. The WHO building blocks domains probed in the interview guide guided the deductive approach. At the same time, we employed an inductive approach to identify and integrate new emerging concepts from the interviews and non-supportive data that did not align with the framework. Codes were compared and added based on the agreement between the two data officers. Four transcripts were coded separately and reviewed together for agreement. In cases of disagreement of codes used, the two officers looked at context the code was applied and agreed on consensus of how to apply the code in other transcripts. The codebook was refined with the new codes and descriptions and used for the rest of the analysis. Coding and data management were conducted in Dedoose (Version 9.0.17) [39], and queries and code co-occurrence outputs were exported to Microsoft Excel to identify themes and quotes associated with specific constructs of the WHO health systems framework. EB and GW reviewed and validated the excerpts and identified themes from recurrent patterns in the data informed by the WHO health systems framework. This qualitative research followed the Consolidated Criteria for Reporting Qualitative research (COREQ) reporting guidelines (Supplement 3) [40].

### Ethics statement

This study obtained ethical approval from the Lesotho National Health Research Ethics Committee (ID 117–2017) and the Harvard Human Research Protection Program (IRB17-19888). Written informed consent was obtained from all participants, and all methods were conducted in accordance with the Declaration of Helsinki.

## Results

### Participants

A total of 21 healthcare workers participated in in-depth interviews, with 10 (48%) conducted at the district level, aligning with the LPHCR’s focus on district-level management. Initially, we intended to conduct 25 interviews across different levels, but a few were not possible due to scheduling conflicts. Of those who couldn’t make it, one central-level interview was discontinued as the participant had competing priorities at the Ministry of Health. Additionally, one interview with a Ministry of Health office representative could not be completed due to inconvenient timing and competing priorities. Participating healthcare workers held various positions, including Director and high-level officials from the Ministry of Health, District Health Managers, Public Health Nurses, Health Center managers (nurse-in-charge), Village Health Worker Coordinators, and Village Health Workers. Refer to Table 1 for participants’ key characteristics.

**Table 1:** Characteristics of participants. LPCHR

Characteristics	Number (%) of participants interviewed
**Level of health system**	
Central Ministry of Health	3 (14%)
District health management office	10 (48%)
Primary care facility	4 (19%)
Community programs	4 (19%)
**Site**	
Berea	5 (24%)
Butha Buthe	6 (29%)
Leribe	3 (14%)
Maseru	3 (14%)
Mohale’s Hoek	4 (19%)
**Sex**	
Female	19 (90%)
Male	2 (10%)

### Predominant themes

The WHO Health Systems Framework guided us in identifying six key themes. These included 1) progressive transformation and improved efficiency in healthcare service delivery, 2) increased health workforce in the health facilities for efficient primary health care services delivery, 3) improved availability and use of data for performance monitoring and decision making, 4) streamlined procurement and distribution processes have significantly reduced stock-outs and improved access to medicine and medical products, 5) strengthened budget management capability of districts, and 6) promoting the institutionalization of district management health teams to facilitate effective leadership and oversight. Themes are presented with illustrative quotes below and in Table 2.

**Table 2:** Themes on HCW perceptions on impact of health reform with supporting quotes

Theme	Example Quotes
Progressive transformation and improved efficiency in healthcare service delivery	*I noticed that the buildings were there already but were not being used. Reforms made sure that they got used.* *(HCW, Berea, Community)*
	*The number of women delivering in the facilities has improved. The antenatal care has improved. For the past 2 to 3 years, we have been awarded as the best-performing district, and we thank the reform for that. We think one of our successes lies in the reform* *(HCW, Butha-Buthe, District)*
	*We can take the whole day talking about achievements, but honestly, health services have improved. Management, and even the clinical part, has improved a lot.* *(HCW, Berea, District)*
	*Ah, besides that, I think the service delivery has improved. Like for example in maternal health, before there were no deliveries that were being conducted. Only eight facilities were conducting deliveries. But now we have everyone [facilities] conducting deliveries.* *(HCW, Leribe, District)*
Increased health workforce in the health facilities for efficient primary health care services delivery	*That just reminds me that it’s a good thing to have a coordinator* *(HCW, Berea, District)*
	*No, in the PIH sites, they were [enough staff]. Everybody knew what their job is and beyond which, when should they refer? And who is responsible? And if there’s a challenge, who do you ask?* *(HCW, Maseru, Central)*
	*We did have a pharmacy and pharmacy technician in the facilities that were of a high population. So it did improve and I think we dropped it, and then some partner picked it up and then it’s working well for them. So having the pharmacy technicians at the facilities, in terms of human resources, it’s helping. And then I think we started it [as part of the] Reform, and then people followed into it and then it’s working so well.* *(HCW, Leribe, District)*
Improved availability and use of data for performance monitoring and decision making	*If you are talking about the health information system, at the community level, actually there are reporting tools which have been developed for the village health workers to use when reporting. And at the facility level, like I have mentioned before, I said that at the community level, we still have the village health workers who are having the tools to report and I also mentioned that those reports or those tools for collecting that information at the facility will be collated by the village health worker supervisors. And I also mentioned that at the facility level, we also have the tools which are being used by the village health worker coordinator. The information from the community level there is collated and then it is delivered to the DHMT, who will then send the information to the central level from the DHMT level* *(HCW, Mohale’s Hoek, District)*
	*I think the reform was supposed to help us analyze our own data properly. Data should be meaningful to its users. Before it was just the numbers. But with the reform, it has now started making sense. Now we have started working smoothly…it can measure our performance, if we are told we have performed well, it is because of the data. So I think the reform has helped a lot* *(HCW, Butha-Buthe, District)*
	*We are able to share our data. We know what is happening in the communities. Most of the time, the facilities they do not report home deliveries, but from the village health worker reports, we are able to know which villages we have home deliveries …and that helps us to act on that. And now we are able to track back our clients who have defaulted.* *(HCW, Leribe, District)*
	*…because they were given the reporting time, I think the completeness has increased. Even the timeliness has increased. I think it’s because they [DHMT] themselves do get the reports in time at their level. So that has changed also.* *(HCW, Mohale’s Hoek, District)*
Streamlined procurement and distribution processes have significantly reduced stock-outs and improved access to medicine and medical products	*Everything was relying on the nurse, supply, ordering they were not even familiar with those steps on how to follow on ordering of the drugs and when some drugs would be coming…while other drugs would expire while they are there, nobody is checking them. He was doing everything, the nurse was doing everything, so that has been a relief for them.* *(HCW, Berea, District)*
	*… But with the Reform, the DHMT and the district health manager were overseeing the entire district. Hence it was our responsibility to share the resources within the district. If one facility or if anything comes to the DHMT and you find that the need is more in a CHAL facility, you don’t say the DHMT gets funding from the government and CHAL gets its funding through the subvention. You just have to see how you will close the gaps. And with the drugs, especially if the donor funds the drugs, it is the responsibility – I am just making an example – of the DHMT to redistribute. If there’s more at Mabote and there’s nothing at Khubetsoana, we pick that up, we take from [one place] to where it is [needed]* *(HCW, Berea, District)*
	*And also the cold chain, the national vaccine store, is within the Ministry, it’s not at the national drug supply. So for all the other commodities that you will get from the National Drug Service Organization, all those are put together. But for cold chain and vaccine management, that one is still done separately though the family health division through the EPI. But at least that’s a reduction of 8 to 2* *(HCW, Maseru, Central)*
Strengthened budget management capability of districts	*So they have, now, also PBF – performance-based financing, which one of the benefits is when you are rolling out PBF in the Reform districts, it becomes very easy because you already have a district health management team that knows their roles and responsibilities. They have their plans and they have already trained their community structure – their structures at the facility level and the community. Whereas, when they were moving, when PBF was moving into a non-Reform, they had to restart everything from training village healthcare workers and community healthcare workers, and some of them, even though they have this additional performance…these Reform districts, once they become PBF, they outperform those ones where there has not been a Reform.* *(HCW, Maseru, Central)*
	*As far as my memory goes, the way I remember the things, we did not see any major change in the funding that we used to receive from the government, because of Reform, or because of now primary healthcare, we have to put more money to the health centers. We did not see it. It was just looking, usually stuff that we used to get previously.* *(HCW, Butha Buthe, District)*
	*There were delays in payments… Serious, like people will be owed for… quite a number of months without getting [paid]. Because there were a lot of verifications and whatever, I don’t know…So what we were told was the government had to take over in the next financial year, hoping they would have budgeted for that. So it didn’t happen. So there were these village health workers that were being paid by PIH, hoping the government would take over. And when that time came, the government did not take over, and people were still expecting their money from Partners In Health. So now the challenge, Partners In Health was saying: “I never budgeted for this much. I had plenty to help until this period, and that time is gone. I can’t continue forever”. So that’s what brought a lot of problems with our village health worker payments.* *(HCW, Berea, District)*
Promoting the institutionalization of district management health teams to facilitate effective leadership and oversight	*“Now we wanted to focus on our own priorities”. Because as I said, if you have a plan – you may have a good plan – but if you don’t have your own priorities you won’t achieve anything. So as a district, we had to say: “What is it that the district needs?” And we put our plans as a priority. It is true that we have to collaborate with our national office. We still have to listen to them and do what they have, but it is not everything that they have for us that is going to benefit our district. Because for them, what they have is cross-cutting across all the districts. So to prioritize, you have to say “Is this going to benefit my district? … If not, do I have to be part of this?” And make sure that I prioritize my plan as long as it is thought towards achieving the Ministry of Health plan – strategic plan – then I should continue with that.* *(HCW, Berea, District)*
	*The DHMTs were formed. All the local doctors had to be recruited looking at their expertise, and then they were the ones who were made to be the DHMTs for those districts. So that was a means of attaining the local people, maybe with expertise, to be there* *(HCW, Butha-Buthe, District)*
	*You cannot give somebody finances who does not have leadership. Would we work? You can give me a hundred thousand, as long as I don’t know what to do with it, you want to see the outcome. But if you give me a hundred thousand and I know what to do with it, then you will see… As I always say, with the six building blocks that we have, leadership and governance is number one. Because as much as leadership and governance are not there, you can give me as many doctors as I can, but if I don’t have leadership and governance, those doctors are going to go up and down and not do their job. Because there’s no leadership and governance… It’s like fertilizer, leadership, and governance. When you are plowing, you have to put in the fertilizer so that everything grows faster.* *(HCW, Leribe, District)*
	*So we said in terms of leadership and governance, it was marvelous.* *(HCW, Leribe, District)*

1. Progressive transformation and improved efficiency in healthcare service delivery

Participants noted initial misconceptions regarding the purpose of the national health reforms in health service delivery, which in some cases resulted in feeling pressure to meet indicators. Participants noted that effective supervision and resultant changes in service delivery dispelled these misconceptions, leading to a more positive outlook on the reforms.

*The nurses felt like they were threatened to start to do deliveries [of babies] but then with the support and the supervision that was done by Partners in Health to the facilities, it made them comfortable to start. So most of them started to conduct the deliveries.*

*(HCW, Berea, District)*

*There has been a difference, there’s continued change annually. You’ll recognize some changes. I believe that at the start [of the reforms], people didn’t know much about them. Even some HCWs at the beginning, might have felt disturbed. However, clinics are now more manageable; they just work well even without DHMT. Everyone can do or perform their different tasks without being pushed knowing it’s their responsibility with clear targets that are driven by them. There is quite an improvement.*

*(HCW, Butha-Buthe, District)*

Many participants reported that the purpose of the reform was to enhance health indicators. For example, community programs and training of village health workers were aimed at facilitating safe deliveries and reducing maternal mortality rates. HCWs reported that the reforms introduced reporting tools and initiatives to raise awareness in the community through *pitsos* (community gatherings).

*The reform was meant to improve our indicators, especially the maternal child health care and the community program in which they assisted us to help the facilities conduct deliveries and save mothers from dying from those complications of labor...We had specific reporting tools that are used by the village health workers that report directly to the district, and they also had some indicators that were planned to improve the maternal child outcomes.*

*(HCW, Butha-Buthe, District)*

A common theme expressed by many HCWs was the improvement in service delivery for health outcomes. One of the participants noted that the reforms resulted in a decline in home deliveries and improved access and utilization of health services. The availability of free services–including medical services as well as supportive services such as food–also contributed to a higher preference for delivering at health centers among women.

*Since the reform, the number of home deliveries has decreased. The health services at the health centers/clinics have improved and this is because of the presence of the coordinator which has brought about good change, when village health workers come to the facility there is always someone to welcome them even when the nurses are busy there is someone ready to receive them. Most women deliver at the health center because it is free of charge and the food is also free of charge, everything is free.*

*(HCW, Berea, Community)*

HCW also noted that expanded service availability facilitated by the reforms brought services to the people and reduced the time required to seek key services.

*It was expensive for patients to travel long distances only to get paracetamol. So now they get it free closer to them at the clinics. There were no deliveries conducted in some of the health facilities, but with the reform all the facilities were assisted or they ensured that all the facilities conducted deliveries so that those women do not have to deliver only at [specific] hospitals.*

*(HCW, Butha-Buthe, District)*

The village health workers (VHW) program, a component of the LPHCR was meant to professionalize and strengthen community health program by formalizing VHW roles and establishing community health information systems. Interestingly, one HCW noted they did not achieve optimal success in the health programs and attributed this gap to difficulties encountered by VHWs who serve as vital intermediaries between the community and healthcare facilities. Some of these challenges included attrition, lack of motivation, and late remuneration payment that led to the sub-optimal success of the VHW linkages between the community and health facilities.

*When we talk about the MDGs [Millennium Development Goals] on service delivery, improving programs e.g. TB, HIV, maternal health, we did not get there [meet target].*

*I think we only got up to sixty, seventy percent. Due to the challenges of village health workers who are key personnel that is actually linking us with the community. They were the key personnel that actually takes the community, which is our clients, to the facility.*

*(HCW, Leribe, District)*

Notably, one HCW's complaint regarding staffing issues resulted in an increase in personnel qualified to conduct deliveries.

*I remember that we complained about the nurses… there was only one or two nurses in the facility – a nurse assistant and a registered midwife. But with the Reform, they negotiated that nurses should be increased in number. So now we have five nurses that can conduct deliveries...there was a movement in the Ministry of Health that they gave us the nurses.*

*(HCW, Butha-Buthe, District)*

The move by the Ministry of Health to increase the healthcare workforce to enhance healthcare services and meet the growing demands of the community was reiterated by many participants.

*Right now, the Ministry has made additional efforts to increase staff at health center level to five. Initially, there were three, but now they have gone up to five. These are the positions that have been recently advertised and filled, and it’s five positions.*

*(HCW, Maseru Central)*

One of the HCWs noted the addition of new staff was a catalyst for positive change in health centers and clinics contributing to enhanced service delivery and overall efficiency.

*The health services at the health centers/clinics have improved and this is because of the presence of the coordinator which has brought about good change, when VHWs come to the facility there is always someone to welcome them even when the nurses are busy there is someone ready to receive them.*

*(HCW, Berea, Community)*

However, some continued challenges in human resources for health, particularly staffing shortages, were also expressed by participants. One HCW expressed a feeling of exhaustion due to the absence of significant improvements in staffing.

*For human resources for health, we are still doing badly. For example, now I would say it’s only two of us. That is why I’m so exhausted, and then I have to wake up in the morning, come here, and see a lot of patients. So for human resource, I don’t think we are there yet because the key personnel for human resource was doctors and nurses and also the village health worker who were going to help us. But we did not see any impact in terms of human resources.*

*(HCW, Leribe, District)*

2. Improved availability and use of data for performance monitoring and decision making

Many participants echoed the positive impact of the national reforms regarding readily available data at the district level. One participant noted the presence of a district health information officer allowed for the efficient collection, systemization, and transmission of data to headquarters, while also enabling local analysis and compilation of monthly district reports for performance evaluation and decision-making.

*Because you already have somebody that is called a district health information officer that was placed at the DHMT, but their job was actually to take this data, put it in the system, and send it to headquarters. That’s it. Then it was changed [with the reforms] to say, you take the data, yes you put it in the system, you send it to headquarters, but again, you deal with it in the district. We see that and then we will say “let’s compile the district report on a monthly basis and then we would look at our report, how we are performing, and how everything is going”.*

*(HCW, Leribe, District)*

One HCW highlighted the significance of available health data in facilitating the tracking and evaluation of the health system's performance, including at the community level. Another participant emphasized how reporting tools not only enabled data collection and transmission to higher authorities but also facilitated the monitoring of community-level performance indicators.

*We are able to trace back even to the community level, how the health system is doing. So it’s a success for me. To know exactly how we fair with our services, what is happening, even at the community level. That is a success. That’s why I said that the reorganization of the program [led to success].*

*(HCW, Mohale's Hoek, District)*

*We were given some templates of reporting…and they were supposed to submit their reports to their supervisors, who would check and verify, then send the reports to the health facility and they will be compiled by the coordinator there who would send that community component to the public health nurse for community. So that really helped a lot, because I think we knew what was happening in the community. And we could also track our performance on the Reform at the community level.*

*(HCW, Berea, District)*

Many participants appreciated the use of the data produced by the health information managers after analysis, which made them see the gaps in service delivery. One of the participants also noted that the reforms resulted in the data being submitted to the Ministry of Health.

*And one of the things that I saw was the reports they make. They are very, very informative. They analyze their data – they know their districts! They know their catchment population. They know how to break it down into, you know…in short, they really are doing a good job.*

*(HCW, Maseru Central)*

3. Streamlined procurement and distribution processes have significantly reduced stock-outs and improved access to medicine and medical products

Many HCWs highlighted the positive transformation in medicine procurement and distribution. Participants noted following the reforms, facilities can procure medicine directly from National Drug Service Organization (NDSO) and the process is smoother. One participant noted patients can now access the medication at the health facilities and there are no stock outs.

*I think we are seeing patients in the health facilities now that they are able to get ARVs, able to get medicine when they are sick. Drugs are always available at the health facilities. That, sort of – in a way has improved – the case, the load of people that are seen in the facilities.*

*(HCW, Butha Buthe, District)*

One of the participants noted that the reforms led to a centralized procurement supply chain system being developed.

*I will give an example of supply chain. When the assessment was done, each of the programs within the Ministry was involved in the procurement of commodities in one way or another. So from the Ministry [of Health], ten different programs were procuring commodities. And one of the suggestions to solve it was there to establish a supply chain unit that could do procurement for all the programs through one source…there was one person that was picked up from all those units to establish a unit, a supply chain unit, interim, so that all the supply is managed through one system. We have managed, currently, to reduce that from 10 to 2.*

*(HCW, Maseru, Central)*

Participants noted there has been a shift in supply provision as a result of the reforms. One of the participants highlighted how previously, the district was dependent on the Ministry. However, with the reforms, a more responsive system was established, where supplies are now provided based on monthly requisitions, ensuring that facilities receive the items they need when requested.

*Of course, since what we are being provided on monthly basis, right? We were dependent from the Ministry [of Health]. We weren’t able to buy some of the supplies like we would need to. But with the Reforms, things changed. These things, the supplies are made on monthly basis, as per requisition, of course. If you aren’t requesting it, you won’t get anything. But if you are requisitioning according to how you use, that one will actually be provided with.*

*(HCW, Mohale’s Hoek, District)*

4. Strengthened budget management capability of districts

Participants reported that the allocation of budgets to the districts is predominantly regulated by the central level. However, in districts where the National Health Reform is implemented and the District Health Management Teams (DHMTs) have been strengthened, there is evident advancement in financial management. One participant observed that the empowered DHMTs are capable of autonomously making decisions at the district level and depend less on the central level, but some authority is limited in terms of allocation.

*And also, what I have also seen, even with the district health management, in the districts where we have the Reform, the DHMT is empowered, they are able to manage their finances well. They are able to… make decisions on their own, at the district level, without relying so much on the central level. They come to central level if there are issues that are not clear – and really need to be implemented.*

*(HCW, Maseru, Central)*

However, a participant raised the concern that there was no observed noticeable increase in funding at the district level to support the allocation of budgets from the central to district level, which was the initial intention of reallocating funds from secondary care to primary healthcare.

*I remember in one of the presentations at the very beginning, what was being said was that Lesotho spends a major amount of money into the secondary level of care, like major health budget is spent at the Tsepong [referral hospital]. So, we wanted to turn that prism upside-down so the money goes to primary healthcare. But that was talk, because at the district level, there was no increase in any funding where we could say, “Okay, previously, I was having this much per project, now my budget has increased to this much”.*

*(HCW, Butha-Buthe, District)*

Additionally, another participant emphasized the challenge of decentralized budget management in the health sector, particularly regarding donor funds. In this context, districts face limited authority in managing funds designated for their use. This lack of decentralized authority for the donor funded was reported as leading to delays, misalignment with their plans, and weakening of district-level implementation.

*But another thing, I think, was also a challenge with our, with – because we have health sector funding. Part of it comes from the recurrent budget. The other funding comes from donors. So our project accounts unit is not decentralized for funding that comes from the treasury, the people in the districts can manage their budget and do everything, once the budget is allocated. But for funding, that is earmarked for districts. As long as it’s from donor projects, it means that even if they have to implement, they still need to send everything to central for implementation of the donor funds. And that also weakens the districts.*

*(HCW, Maseru, Central)*

5. Promoting the institutionalization of district management health teams to facilitate effective leadership and oversight.

Participants were generally in favor of the changes in leadership and governance as a result of the reforms. One participant from Leribe District noted, “*I said the things that I can write home about the reforms, it’s the leadership…*”. Many participants felt the DHMTs were revitalized and granted authority to actively contribute to the management of their districts. Health centers committees were also revived and allowed communities to become integral to healthcare delivery, enabling them to have a say in how healthcare services are provided to them, making community participation a tangible reality.

*Previously, DHMT was just concentrating on primary healthcare. With Reform, DHMT is the senior management of the district, running both curative and primary healthcare.*

*(HCW, Leribe, District)*

Participants also noted that the leadership skills and understanding of roles among the health center committees have significantly improved through mentoring, supervision, by the DHMT and experience. One of the participants highlighted how post reform they have become more effective in managing patients and the overall health system, leading to enhanced healthcare delivery at the health centers.

*In terms of leadership, and understanding their roles, I think they are much better. They have gained a lot from that with experience because we support them with mentoring and supervision. They are better off. They manage patients better. They manage the health system – the health centers better.*

*(HCW, Berea, District)*

One of the HCWs also reported how supervision improved following the reforms and a hierarchical leadership structure, which includes different levels of authority and responsibility established.

*Supervision was not going well at all. (Laughs). Yes. Supervision was not going well at all, at all levels from district, health center, district to health center, health center to village, that was not going well at all. And it improved, as a result of the Reform. Like I told you, now we have a supervisor. That is something new. We didn’t have anybody called “village health worker supervisor”.*

*(HCW, Maseru, Central)*

Many participants overall felt the LPHCR had been successful in improving and revitalizing primary healthcare and district health systems, particularly in terms of leadership and governance. A participant from Leribe highlighted how other districts have even begun to emulate the practices implemented in the Reform, demonstrating its positive impact.

*In closing, what I can say is actually, Reform was a good initiative, that I think – if it was supported by central governance – it was going to succeed and it was actually going to improve and revitalize our primary healthcare, even the health systems at the district. As we have seen, it actually achieved a hundred percent in leadership and governance. That one is okay because the other districts are actually copying from us, as a district that are practicing Reform. So in terms of leadership and governance, we’re there.*

*(HCW, Leribe, District)*

## Discussion

Our study presented the perspectives of health workers on the decentralization of health care management to the district level in four pilot districts as part of LPHCR. The findings indicated that those interviewed felt that there were significant improvements in primary health service delivery due to increased investment in human resources for health, enhanced availability and utilization of data for performance monitoring and decision-making, streamlined procurement of medicines and medical supplies resulting in fewer stockouts. Those interviewed believed that these elements of health care delivery were tied to improved health program management and effective leadership through district health management teams being empowered to respond directly to challenges. The results of this study have important implications for policymakers and healthcare managers in Lesotho and other countries that have implemented decentralization policies to improve their health systems. We observed some variations in districts' decisions about optimal allocation and utilization of financial and human resources. Fostering local autonomy in decision-making by enabling all districts to lead primary health care initiatives could strengthen the core elements of the health system, promote efficiency and effective problem-solving while addressing inequities in health care delivery.

Similar studies conducted in LMICs suggest that the effectiveness of facility and district managers in settings with high autonomy introduced through decentralization is closely tied to control over resources and adequate management skills, which led to better service delivery and health outcomes [5, 41]. A study in Zambia found that decentralization facilitated districts in making decisions regarding resource allocation, aligning with our findings of districts having autonomy in budgeting and financial management [42, 43]. In line with our study, a quantitative study examining the impact of the LPHCR on health service delivery building block demonstrated significant improvements in primary health care service delivery and healthcare utilization across the four pilot districts following the decentralized model [28]. Qualitative interviews further supported this quantitative finding, highlighting the role of organized leadership, improved reporting tools, and the introduction of an audit and feedback strategy through the LPHCR as catalysts for the observed changes. Systematic reviews conducted in 2017 on the decentralization of health systems in LMICs have demonstrated positive effects on the system, although our study indicates challenges in resource management [44].

Despite the decentralization of management to the district level, challenges remain in decision-making autonomy, budget management at health facilities, and funding availability, impeding the achievement of reform goals. Similar challenges are observed in other countries, attributed to factors like lack of political commitment, insufficient resources, and technical capacity at the district or regional levels [5, 45–47]. These obstacles have significant implications for health system effectiveness and sustainability of the decentralized model. Limited autonomy impedes tailored service delivery, while inadequate budget management leads to disparities and compromised care quality. Addressing these challenges requires political commitment, adequate resources, capacity-building, and effective governance to fully leverage the potential of decentralization in enhancing health system performance [48].

### Strengths and limitations

Our study provides a focus on healthcare workers' experiences at the facility, community as well as district level, providing a better understanding of their perspectives and experience in national PHC reforms. The results addressed a gap in the literature and contributed to the ongoing debate about the role of decentralization in improving healthcare delivery in LMICs from the healthcare workers' point of view. The use of qualitative interviews added richness to our understanding of the research question and provided insights into the experiences of healthcare workers regarding the reforms. To minimize social desirability bias, skilled qualitative interviewers who were trained in probing techniques conducted the interviews in a private space consented by the respondents, maintaining confidentiality. Participants were assured that their personal information would be kept confidential to prevent any adverse employment consequences should they be identified as criticizing their workplace or performing poorly, hence they were open to provide information objectively both positive and negative.

Our study faced limitations related to the relatively small sample size of healthcare workers at different health care levels, primarily due to the complexities of implementing the intervention across different levels of the health system and the resource-intensive nature of data collection. As a result, we conducted interviews with willing participants, excluding some senior directors from the central Ministry of Health due to scheduling conflicts. Additionally, about half of the interviews were sourced from the district level, potentially introducing a selection bias. Nevertheless, these interviews effectively captured the experiences and opinions of the healthcare workers involved, aligning with earlier quantitative studies focusing on district health management. There is a possibility that we missed identifying less frequent yet potentially important themes at some of the health care levels presented by participants. However, the size of our study was adequate to achieve saturation for themes across the interviews [49]. To gain more comprehensive insights, future research should include feedback from other sectors and stakeholders. This would provide a deeper understanding of the impact of decentralization on healthcare systems. Despite these limitations, our study contributes valuable insights and highlights the need for further research to address the identified limitations and explore the perspectives of top leadership and broader stakeholder groups. By considering these recommendations, a more comprehensive understanding of the impact of decentralization can be achieved. We had planned to use certain ideas and theories from the implementation science field, but we ended up using a different framework called the WHO health system building blocks. The decision not to conduct this assessment as implementation research was determined by the initial design, which focused on health system building blocks. In the analysis, the data collection tool and the proposed analysis didn't align with most implementation research analysis frameworks.

## Conclusions

This paper contributes to the series of studies on the Lesotho Primary Health Care Reform (LPHCR) and its impact on the healthcare system. The study highlights the positive effects of decentralization on service delivery and healthcare utilization, while also identifying areas for improvement. Decentralization empowered district leaders and enhanced the responsiveness of healthcare services by granting decision-making authority and autonomy. This authority equipped and instilled confidence in the district health management team, enabling them to provide effective primary healthcare in their respective districts. However, challenges such as limited decision-making autonomy, budget management constraints, and funding availability bottlenecks still need to be addressed to fully realize the potential of decentralization. Our findings align with the growing evidence on the positive impact of decentralization in LMICs, highlighting the need for ongoing efforts to document frontline implementers' perspectives for informed health policy development. Continued investment and support, including political commitment, adequate resources, capacity-building, and effective governance structures, are crucial for successful decentralization across Lesotho's districts. This will lead to improved healthcare access, quality, and equity, ultimately benefiting the population's health outcomes.

### Supplementary Information

Supplementary Material 1.

Supplementary Material 2.

Supplementary Material 3.

## Data Availability

The data supporting this work are available in the article’s text and as a table in the supplementary file.

## Abbreviations

DHMT: District Health Management Team
LMICs: Low-to-middle income countries
LPHCR: Lesotho Primary Health Care Reform
MoH: Ministry of Health
PIH: Partners In Health
WHO: World Health Organization
