# Supplementary material for: Healthcare workers’ views on decentralized primary health care management in Lesotho: a qualitative study
Source: BMC Health Serv Res. 2024 Jul 11;24:801. doi: 10.1186/s12913-024-11279-3 (PMC11241925; doi:10.1186/s12913-024-11279-3)

Supplement 1.1: Management and Leadership at the District Level Before Lesotho Primary Health Care Reform (LPHCR) Source: District Health Management Team (DHMT) interview, Director of Primary Healthcare (PHC), Ministry of Health (MoH) interviews. DMO refers to the District Medical Officer. HR officer is the Human Resources Officer. Figure source: Matheson Consulting.

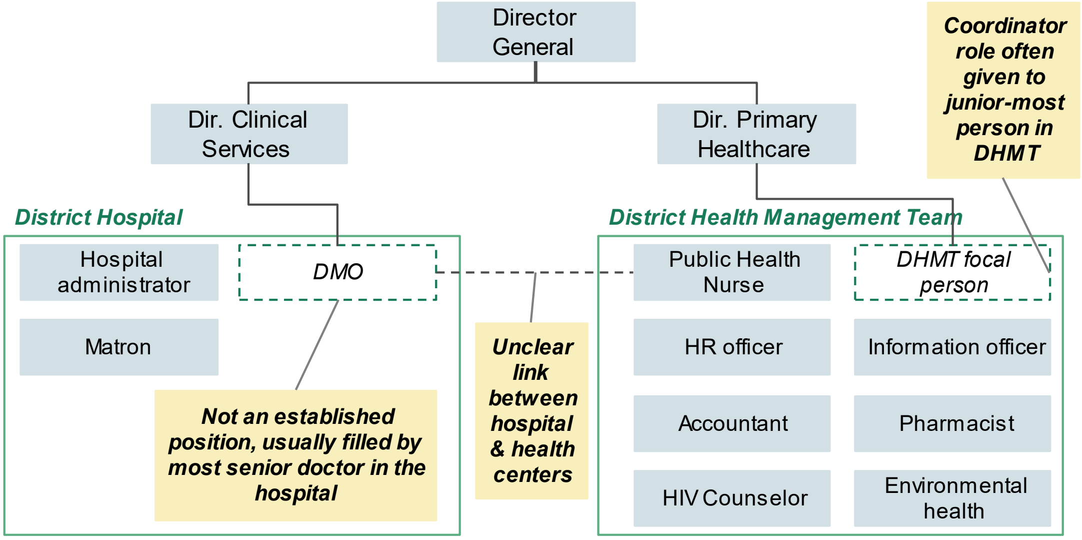

Supplement 1.2: LPHCR introduced organizational structure. The orange area indicates the reorganization guided by the MoH PHC team. QA stands for quality assurance, M&E stands for Monitoring and Evaluation, PHN refers to Public Health Nurse, and VHW represents Village Health Worker. Figure source: Matheson Consulting and Partners in Health Lesotho.

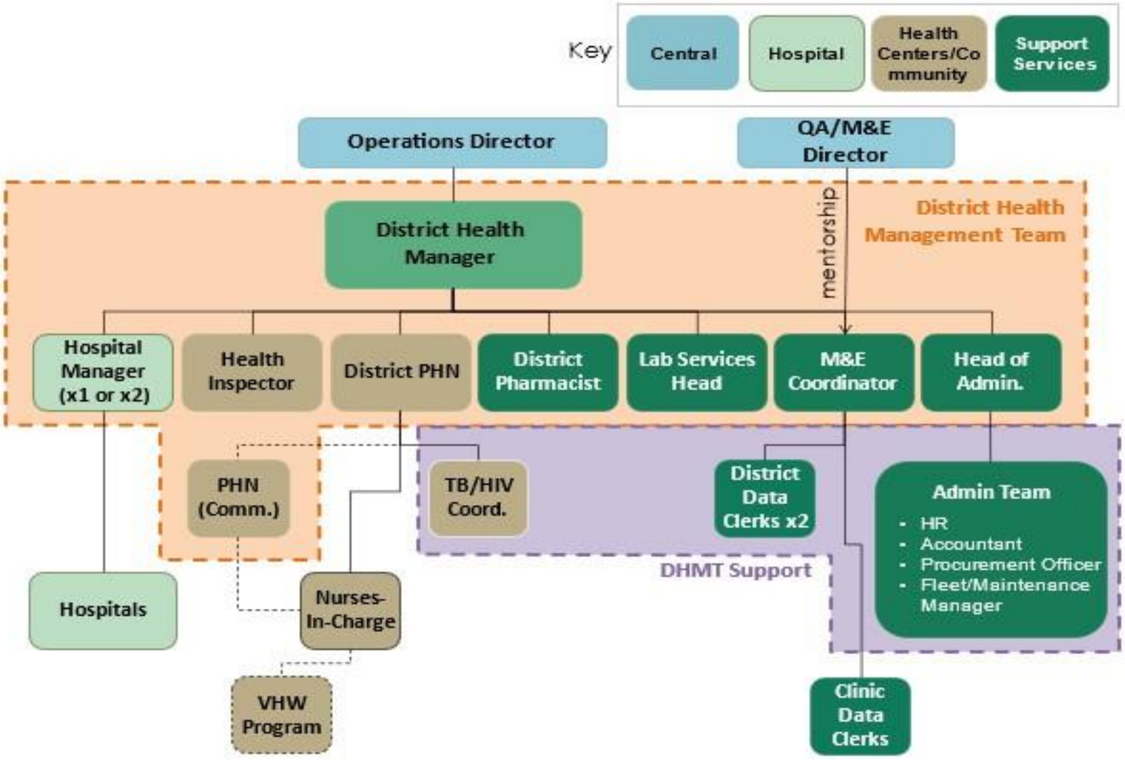

Supplement: Supplementary file 1 — Supplementary Material 1. [file 12913_2024_11279_MOESM1_ESM.pdf]
