# Supplementary material for: Healthcare workers’ views on decentralized primary health care management in Lesotho: a qualitative study
Source: BMC Health Serv Res. 2024 Jul 11;24:801. doi: 10.1186/s12913-024-11279-3 (PMC11241925; doi:10.1186/s12913-024-11279-3)
Supplement: Supplementary file 2 — Supplementary Material 2. [file 12913_2024_11279_MOESM2_ESM.pdf]

Script for Reform User Guide Interviews
8 Aug 2017

KEY INFORMANT INTERVIEW GUIDE

DISCUSSION OF REFORM KEY COMPONENTS

INTRODUCTION

We are here today so that you can share your insight into the *design* and *purposes* of the Lesotho National Primary Healthcare Reform – the effort to improve the healthcare delivery system throughout the country. This discussion will include several specific questions, but I want you to feel comfortable sharing information from your knowledge of the Reform. Any views expressed today will be kept private—neither your name nor any identifiable information will be shared with anyone, though we do plan to convey the insights of everyone we interview anonymously in order to guide future reform efforts. I am not a member of the Government; I am an employee of Partners In Health, a healthcare delivery organization in Lesotho.

The goal of our project is to understand the model of the National Primary Healthcare Reform. More specifically, for each component of the health system, we would like to understand what changes the Reform was *supposed* to introduce, if any. There will be a separate set of interviews that will focus on the implementation of the Reform, including what has gone well and what has not gone well to-date. However, the purpose of today's interview is to document the *initial goals* of the overall project rather than the process of its implementation so far.

In order to understand this, we need to talk with people who have been involved in designing the Reform—which is why it is so important that you are here today. There are no right or wrong answers to the questions in this interview, so we are asking that you actively contribute inputs from your knowledge. We will be recording this conversation, but this recording will only be used to remember what we have discussed later. Your responses will be kept confidential, so please speak freely. Do you have any questions before we begin?

Facilitator Checklist

Mention all of the following:

- Your name
- Goal of project
- Work at Partners In Health
- Do not work for MoH
- Confidentiality
- No right or wrong answers
- Use of audio recorder
- Ask if any questions

Script for Reform User Guide Interviews
8 Aug 2017

PART 1: WARM-UP

1. Let's start with you telling me a little about yourself: Your name, as well as where you are from and a little about your background and current job.

2. How have you been involved in the National Primary Healthcare Reform efforts?
   Note: If any of the following topics DON'T come up during discussion, probe further.
   a. With what institution(s) have you been involved in the Reform? Where?
   b. What role(s) have you played within that institution in relation to the Reform?
   c. When were you involved in these efforts? If in the past, when?
   Note: If the respondent has worked in multiple organizations or roles, probe further to ensure that the above questions are answered for every role/organization/area they have worked in.

[TRANSITION] Thank you. Now that I have an understanding of your background, expertise and role in the Reform, I'd like to ask some descriptive questions—just so we can better understand the Reform from your perspective.

PART 2: HISTORICAL OVERVIEW

1. Could you describe, as best as you can, your understanding of what the Health Reform is supposed to achieve for Lesotho? I know this is a big question, so feel free to take your time.

2. Are there aspects of the Reform that are not clear to you? If so, can you tell me more about what aspects are unclear?

[TRANSITION] Ok, thank you. Let's take a moment to talk a bit more about some specific areas of the health system. For each of these areas, it would be helpful to know – from *your* perspective on the Reform – what was supposed to happen, what has happened, and what still needs to be done. Because this next section covers a lot of material, you may not know the answer to some of the questions. If this is the case, please feel comfortable saying "I don't know."

Script for Reform User Guide Interviews
8 Aug 2017

PART 3: SERVICE DELIVERY

1. Was the Reform supposed to introduce any changes to the types of primary healthcare services that are publically available? If so, can you tell me about these changes?
   Note: if the following areas are not mentioned, probe further – *How about for…?*
   a. Outpatient department visits (OPD)
   b. HIV
   c. Tuberculosis (TB)
   d. Maternal-child health (MCH)
   e. Non-communicable diseases (NCDs)
   f. Community-based services

2. Was the Reform intended to change the ways that people use health services? If so, how?

3. Was the Reform intended to change the quality of health services? If so, how?

4. Was the Reform supposed to change anything about the way that performance of the health system is monitored? If so, can you tell me a bit more about this?

5. Okay, thank you. With the Reform, were there supposed to be any changes in the way that care is managed for patients? *For instance, were there supposed to be any changes in the management of care for patients who have both TB and HIV, or for patients who need referrals to higher levels of care?* If so, what was supposed to change?

6. Was the Reform supposed to introduce any changes to infrastructure in the health system, such as new facilities, new types of facilities, or updated facilities? If so, can you tell me more?

7. Are there any other ways that health services were supposed to change with the Reform that we haven't discussed yet? If so, please tell me more about these.

[TRANSITION] Okay, thank you. Let's move on and talk a bit about the people who are providing those services… the health workforce.

PART 4: HEALTH WORKFORCE

1. Was the Reform supposed to change the number of health providers working at the community, facility, or district levels? If so, how?
   Note: if the following areas are not mentioned, probe further – *How about for…?*
   i. Nurses
   ii. Village health workers, VHW supervisors, and VHW coordinators
   iii. Pharmacy & lab staff - pharmacist, pharmacy technician, lab technicians
   iv. Other personnel

2. And how about the roles served by health providers? Was the Reform intended to change the roles of health providers working at the community, facility, or district levels?
   Note: if the following areas are not mentioned, probe further – *How about for…?*
   i. Nurses
   ii. Village health workers, VHW supervisors, and VHW coordinators

iii. Pharmacy & lab staff - pharmacist, pharmacy technician, lab technicians
iv. Other personnel

3. Okay, thank you. Was the Reform supposed to introduce any changes in the way that staffing gaps are monitored and filled? If so, what changes were supposed to occur?

4. Was the Reform supposed to change anything about the way that health workers receive ongoing training (e.g. through workshops)? If so, can you tell me more about this?

5. Are there any other ways that the Reform was supposed to change the health workforce that you haven't mentioned yet, but would like to? If so, what are these?

[TRANSITION] Okay, great. Let's transition again and talk a bit about leadership in the health system.

PART 5: LEADERSHIP & GOVERNANCE

1. When the Reform was introduced, was anything supposed to change in terms of the roles served by the central MOH, DHMTs, facilities, or local governments? If so, how were roles supposed to change?

2. Was the Reform intended to change anything about the way that problems in the health sector are solved? If so, can you tell me more?

3. Was the Reform supposed to introduce any changes to health policies, such as national health strategies or policies for specific issues (e.g. TB, HIV/AIDS, or maternal-child health)? If so, can you tell me more?

4. Are there any other ways in which the Reform was supposed to change the leadership structure of the health system that we haven't discussed yet? If so, what are these?

[TRANSITION] Okay, thank you. Moving on, let's talk about the way that money is managed within the health system.

PART 6: FINANCING

1. Was the Reform supposed to change anything about the management of funds at the health center, district, or national levels? If so, can you tell me more?

2. Was the Reform supposed to change anything about the way that health providers – such as nurses, village health workers, or others – are paid? If so, what?

3. Was the Reform intended to introduce any changes in the amount of money spent on health at the facility, district or national levels? If so, what was supposed to change?
   Note: if the following areas are not mentioned, probe further:
   a. How about changes in the budget utilization rate? Was the Reform supposed to introduce any change in the percentage of the budget that is used each year? If so, what?
   b. What about changes in the distribution of money – was the Reform supposed to introduce any changes in the areas where money is spent on health? If so, what?

4. Was the Reform supposed to introduce any changes in the system for tracking the flow of finances from one place to another? If so, what changes were supposed to take place?

5. Are there any other changes to health financing that were supposed to occur that we haven't discussed yet? If so, what are these?

[TRANSITION] Okay, great. Let's shift focuses and talk about the system for managing health-related information.

PART 7: HEALTH INFORMATION SYSTEMS

1. *Data generation*: Was the Reform supposed to change anything about the way that health-related data are produced or managed? If so, how?
   Note: if the following areas are not mentioned, probe further:
   a. Was the Reform supposed to introduce any changes in the quality or availability of data? If so, what?
   b. Was the Reform supposed to change the protocols for tracking patient-level outcomes or facility-level reports? If so, how?
   c. Was the Reform supposed to introduce any changes to the forms, manuals, computers, or internet at health facilities or district offices? If so, what?

2. *Compilation, Analysis, and Synthesis*: Was the Reform intended to change anything about the way that health-related data are analyzed? If so, can you tell me more?
   Note: if the following areas are not mentioned, probe further:
   a. Was the Reform supposed to alter the way that data is communicated between levels of the health system – *for instance, between health centers and the DHMT?* If so, how?
   b. Was the Reform supposed to introduce any changes to the ways that health-related data are analyzed? If so, what?
   c. Was the Reform supposed to change anything about the roles or responsibilities of those tasked with conducting monitoring and evaluation? If so, what?

3. *Communication and Usage*: Was the Reform supposed change anything about the way data are used *(for instance, to inform decision-making)?* If so, can you describe these changes?

4. Are there any other ways in which the Reform was supposed to change the health information system that we have not discussed yet? If so, what are these?

[TRANSITION] Okay, thank you. We have one more section to cover, which relates to medicines and medical products.

PART 8: AVAILABILITY OF MEDICINES AND MEDICAL PRODUCTS

1. Was the Reform supposed to introduce any changes in the way that essential medicines or medical products are procured? If so, what are these changes?

2. How about distribution – was the Reform supposed to change anything about the way that medicines or medical products are supplied to health centers? *For instance, equipment, infection control materials, or laboratory materials?* If so, what?

3. Was the Reform intended to change the frequency or duration of medication stock-outs? If so, how?
    a. Was the Reform supposed to introduce any new treatment regimens or medical equipment? If so, what?

4. Was the Reform supposed to introduce any changes to public health efforts – such as new outreaches or vaccination efforts? If so, can you tell me more?

5. Was the Reform intended to introduce any other changes to the availability of medicines or medical products? If so what were these?

PART 9: FINAL THOUGHTS

1. Great, thank you so much for your time. Before we wrap up, I'm curious if—based on all of our discussion—you have any final reflections you would like to share?

2. As a last request, do you have any historical documentation—including guidelines, protocols, or manuals—that have supported the planning or implementation of the Reform? If so, are you willing to share a copy with me?

3. Okay, great. And one last thing: Do you have any questions for me that I can answer?
